# Acute renal involvement in organophosphate poisoning: histological and immunochemical investigations

**DOI:** 10.1080/0886022X.2018.1489289

**Published:** 2018-07-17

**Authors:** Yasemin Kaya, Orhan Bas, Hatice Hanci, Soner Cankaya, Ismail Nalbant, Ersan Odaci, Hüseyin Avni Uydu, Ali Aslan

**Affiliations:** a Department of Internal Medicine, Faculty of Medicine, Ordu University, Ordu, Turkey;; b Department of Anatomy, Faculty of Medicine, Ordu University, Ordu, Turkey;; c Department of Histology and Embryology, Faculty of Medicine, Karadeniz Technical University, Trabzon, Turkey;; d Department of Biostatistics, Faculty of Medicine, Ordu University, Ordu, Turkey;; e Department of Urology, Faculty of Medicine, Ordu University, Ordu, Turkey;; f Department of Histology and Embryology, Faculty of Medicine, Karadeniz Technical University, Trabzon, Turkey;; g Department of Medical Biochemistry, Faculty of Medicine, Recep Tayyip Erdogan University, Rize, Turkey;; h Department of Physiology, Faculty of Medicine, Ordu University, Ordu, Turkey

**Keywords:** Organophosphate, fenthion, renal injury, poisoning, histopathological investigation

## Abstract

**Purpose:** Today, the long-term effects of partial exposure of cholinesterase on the kidney continue to be a research topic. In this study, we aimed to histopathologically investigate the possible effect of acute toxicity due to fenthion, an organophosphate (OP) compound, on the kidneys.

**Methods:** In all, 21 rats were randomly divided into three groups. Experimental group was each administered intraperitoneal 0.8 g/kg fenthion within physiologic serum. Sham group was only administered intraperitoneal physiologic serum. The control group continued normal nutrition with no procedure performed. After 24 h, all rats were sacrificed by cervical dislocation. Half of the recipient kidney tissues were examined histopathologically and the other half biochemically.

**Results:** No histopathological findings were found in the control group. Rats in the experimental group were observed to have epithelial cell disorganization in tubules, moderate epithelial cell loss, and degeneration. Again, expansion of tubules, vacuolization of tubular epithelial cells, and tubular structure approaching atrophy were observed, with cells approaching apoptosis and common hemorrhage noted although rats in the sham group were observed to have mild tubular degeneration.

**Conclusions:** It should not be forgotten that one of the causes of systemic complaints linked to acute toxicity exposed to the OP compound of fenthion may be cellular injury to glomerular and tubular structures in the kidneys.

## Introduction

Organophosphate (OP) derivative intoxication has become one of the most significant public health problems globally in the last 60 years. According to World Health Organization estimates, each year more than three million OP derivative intoxication cases occur. Of these more than 250,000 will result in death. Globally, 30% of suicides occur by taking OP derivatives [1–4].

The most important mechanism of OP toxicity is irreversible inhibition of cholinesterase enzyme. Therefore, neurotransmitter acetylcholine accumulates in the synaptic gap, causing overstimulation of nicotinic and muscarinic receptor in the central and peripheral nervous systems [5–7]. Cholinergic overstimulation leads to the symptoms and complications such as salivation, lacrimation, exophthalmos, diarrhea, miosis, hypothermia, muscle fasciculations, bradycardia, bronchospasm, neuromuscular function changes, pulmonary edema, pneumonia, pancreatitis, and renal failure. Stimulation of the nicotinic and muscarinic receptors causes central respiratory depression, agitation, seizures, and coma [6–9].

Fenthion is one of the OP insecticides most widely used for control of many varieties of insects in agriculture and public health [10]. Various studies suggested that both renal circulation and electrolyte excretion were under partial cholinergic control so that partial exposure to cholinesterase may disrupt those renal functions. Also it has been shown that OP poisoning often led to pathophysiological damage in the kidney [7–12]. In this study, we aimed to histopathologically investigate the possible effect of acute toxicity due to fenthion, an OP compound, on the kidneys.

## Methods

### Animals and ethical procedures

Rats were supplied from Karadeniz Technical University, Experimental Animals Surgical Research Center. The study procedure was approved with 16/02/2017 dated and 82678388/02 numbered decision by Ordu University, Experimental Animals Local Ethics Committee. During the study, all experimental and surgical applications were carried out following the Guide to the Care and Use of Experimental Animals published by the US National Health Institutes, and considering ethics principles.

### Experimental procedures

A total of 21 Wistar-Albino female rats weighing between 180 and 230 g were included in the study. After adaptation period of 1 week in Ordu University, Experimental Animals Research Center, experimental procedures were initiated. The rats were divided into three groups as experimental, control, and sham groups with seven rats in each group. All rats were subjected to standard feeding in the feeding and care room at 22 ± 2 °C temperature and 50%** **±10 humidity under 12 h of light/dark cycle. Rats in the experimental group were each administered intraperitoneal 0.8 g/kg fenthion (Lebaycid, Bayer Crop Science, East Hawthorn, Australia) within physiologic serum. Rats in the sham group were only administered intraperitoneal physiologic serum. Rats in the control group continued normal nutrition with no procedure performed.

### Tissue preparation procedures

The rats were sacrificed with cervical dislocation (Ketalar VR 50 mg/kg, Eczacıbaşı, Istanbul, Turkey) under anesthesia 24 h after the experimental procedures. Abdomens of the rats were opened to access the kidneys. The kidney tissues collected were divided into two parts from the middle, half of them were numbered and put into glass bottles containing 10% neutral formalin for the histopathologic examination. The other half of the tissues were put into Eppendorf tubes for biochemical procedures and kept in the deep freezer at −80 °C.

### Light microscopic procedures

The kidney tissues fixed in 10% neutral formalin, and were than subjected to routine tissue processes. The tissues were embedded in the paraffin blocks after the processes. Sections of 7 μm thickness were cut using rotary microtome (RM 2255; Leica Instruments, Nussloch, Germany). The tissue samples were stained with hematoxylin–eosin (H&E) and periodic acid Schiff (PAS). The evaluation of the stained tissues was performed under a light microscope (BX51, Olympus, Tokyo, Japan) with the guidance of a histologist. The images of the examined tissues were acquired.

### Biochemical tissue analysis

The following procedures were carried out to determine the levels of malondialdehyde (MDA) and glutathione (GSH) in the kidney tissues: The tissues were mixed with fluid nitrogen to prepare the tissue homogenate. Then, 0.5 g tissue was processed with an appropriate buffer of 4.5 mL (GSH: pH, 7.4/50 mM Tris-HCl buffer, lipid peroxidation (LPO): 10% KCl solution). The tissue mixture was homogenized for 15 min (Ultra Turrax T25; Rose Scientific Ltd, Edmonton, Canada). The homogenates were filtered and centrifuged at 4 °C. The supernatants formed were used in biochemical processes. The biochemical processes were carried out using UV–Vis spectrophotometer (UV-1601; Shimadzu Co., Kyoto, Japan). LPO levels and MDA levels were measured in the kidney using thiobarbituric acid test [13]. The results were expressed as nmol/g tissue [13]. GSH levels were measured using 5,50-dithiobis (2-nitrobenzoic acid) and following the method described by Sedlak and Lindsay [14]. The absorption was measured at 412 nm and the tissue GSH levels were expressed as nmol/mg tissue [14].

### Statistical analysis

The statistical analyses were performed utilizing SPSS (Statistical Package for Social Sciences) for Windows v. 20.0. In the evaluation of the data, mean and standard deviation were used for the descriptive statistics. Mann–Whitney U**-**test was used in comparison of the groups. The results were evaluated in 95% confidence interval, and the significance was set at *p* < .05.

## Results

### Histopathological findings

#### Histologic examinations performed in the sections stained with H&E

##### Control group

At the end of the study, the cortex and medulla distinction could be observed in control group rats, with kidney tissue with normal structure glomerulus and tubules observed and no histopathological findings encountered.

##### Experimental group

Rats in the experimental group were observed to have epithelial cell disorganization in tubules, moderate epithelial cell loss, and degeneration. Again, expansion of tubules, vacuolization of tubular epithelial cells, and tubular structure approaching atrophy were observed, with cells approaching apoptosis and common hemorrhage noted. Among other noteworthy findings were atrophic glomerular structures, nerve loss between visceral and parietal leaves in the Bowman capsule, and severe hemorrhage between collecting tubules in the medullar region.

##### Sham group

**Figure 1.**
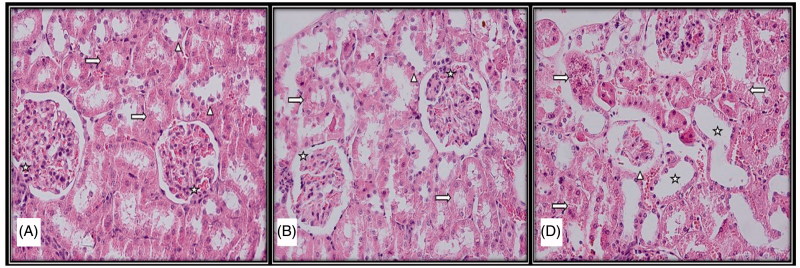
Light microscopic micrographs of rat kidney sections stained with H&E from control (A), sham (B), and experimental (D) groups. (A) Structure of kidney glomerular (star), proximal (right arrow) and distal tubule (arrowhead) with normal histological structure in the histological sections of the control group. (B) Structure of kidney glomerular (star), proximal (right arrow), and distal tubule (arrowhead) with normal histological structure in the histological sections of the sham group. (D) Atrophic glomeruli (arrowhead), tubular enlargement (star), disorganization in proximal and distal tubular epithelial cells (right arrow), tubular epithelial cell loss (left arrow).

Although rats in the sham group were observed to have mild tubular degeneration, kidney tissue with generally normal histological structure and similar to the control group was observed (Figure 1).

#### Histologic examinations performed in the sections stained with ASC PAS

##### Control group

The brush borders with microvillus located in the apical surfaces of the cells that form the proximal tubules found in the cortex of the organ, which had a normal histologic structure were found to be PAS (+). It was also observed that parietal leaf of the Bowman capsule and basal laminas of the capillaries forming the glomerulus had a normal structure and had PAS (+).

##### Sham group

A few PAS (+) granules were observed in the cytoplasm of the proximal tubule cells; however, in general, the kidney tissue was similar to that of the control group.

##### Experimental group

**Figure 2.**
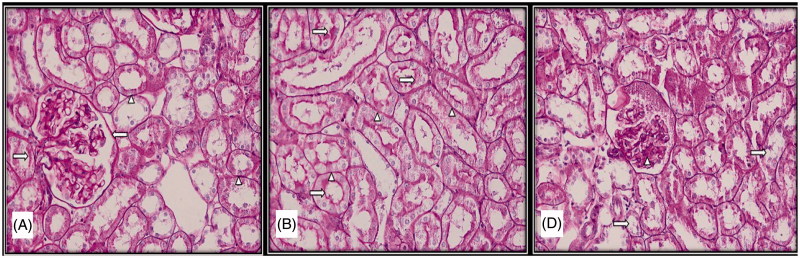
Light microscopic micrographs of rat kidney sections stained with PAS from control (A), sham (B), and experimental (D) rats. (A) Bowman capsule (left arrow), proximal and distal tubule basement membranes (arrowhead) and proximal tubule bristle edge structure (right arrow) of kidney glomeruli with normal histological structure in the histological sections of the control group. (B) Proximal and distal tubule basement membranes (arrowhead) and proximal tubule bristle edge structure (right arrow) with normal histological structure in the histological sections of the sham group. (D) Brush edge loss (right arrow) and dense PAS (+) mesangial cells (arrowhead) in the proximal tubule epithelium.

Intensive presence of PAS**-**positive cytoplasmic granules was evident in the cytoplasm of the cells forming the proximal tubules. Brush border structure with impaired continuity and losses in the proximal tubule cells was among the observed findings. In addition, it was found that parietal leaf of the Bowman capsule of the glomerulus which was remarkable with the thickenings, basal laminas of the capillaries, and mesangial cell cytoplasms were intensively PAS (+) (Figure 2).

### Biochemical analysis results

According to the results of MDA and GSH analyses, MDA level was significantly higher in the experimental group (5.81 ± 2.35 µmol/g tissue) compared to the Sham group (3.18 ± 0.95 µmol/g tissue) and the control group (3.48 ± 0.96 µmol/g tissue) (experimental group–control group, *p* = .037; experimental group–Sham group, *p* =.003). GSH level was significantly higher in the experimental group (1.93 ± 0.20 µmol/g tissue) compared to the Sham group (1.15 ± 0.17 µmol/g tissue) and the control group (1.43 ± 0.07 µmol/g tissue) (experimental group–control group, *p* = .020; experimental group–Sham group, *p* = .010) (Tables 1 and 2).

**Table1.** MDA and GSH levels in tissues.

	MDA (µmol/g tissue)			GSH (µmol/g tissue)		
	Control	Sham	Experiment	Control	Sham	Experiment
Kidney	3.48 ± 0.96	3.18 ± 0.95	5.81 ± 2.35	1.43 ± 0.07	1.15 ± 0.17	1.93 ± 0.20

MDA: malondialdehyde, GSH: glutathione.

**Table 2.** Comparison of GSH and MDA levels between groups.

	GSH	*p* value	MDA	*p* value
Kidney	E-C	.037	E-C	.020
	E-Sh	.003	E-Sh	.010

E: experiment, C: control, Sh: sham, MDA: malondialdehyde, GSH: glutathione.

## Discussion

In this study, we found degeneration in the tubule epithelial cells and epithelial cell loss, and atrophy in the glomerular structures at histopathological level in kidney exposed to fenthion. In addition, we found increases in the level of MDA, which reveals as a result of the lipid oxidation, and in the level of GSH, which is a peptide with antioxidant effect.

Deaths from acute OP intoxication are usually resulted from the depression of the respiratory system of the central nervous system, neuromuscular weakness, and respiratory failure caused by a combination of excessive respiratory secretions and bronchoconstriction. Furthermore, cardiovascular collapse and vasodilatation also contribute to this process [15]. The mortality rate may reach 40% despite sufficient medical care in well-equipped intensive care units [4] Therefore, it has been forbidden in the United States and Canada. However, it is still being produced in several countries such as China and India, and its use as an insecticide is continuing in some countries including Nigeria [16].

Besides the essential function of the inhibition of acetylcholinesterase enzyme, OP compounds have other functions such as hormonal, neurotransmitter, and neurotrophic impacts. In addition, these compounds contribute to inflammatory changes through the enzymes associated with beta**-**amyloid protein metabolism. It has been reported that, with this mechanism of action, they may cause negative effects on different systems such as acute respiratory failure, hepatotoxicity, neurotoxicity, genetic toxicity, embryonal toxicity, immunotoxicity, pancreatitis, hypoglycemia, increased salivation, convulsion, and orchitis [17,18]. Diazinon, orten, malathion, parathion, chlorpyrifos, quinalphos (ekalux), sarin, dimethoate, acephate, and dichlorvos are among the organic phosphorous compound of phorate and fenthion [19–24]. Nephrotoxic effects of some of these compounds have been reported in the publications [24–27].

Acute renal failure is one of the problems which is manifested in clinical follow-up of the patients and cause increase in mortality in OP intoxication [9,28]. In a study, the risk of development of acute renal failure has been reported to be higher by 6.17 times in patients exposed to OP (4). Although various mechanisms have been proposed for the development of acute kidney failure in OPs intoxication, knowledge on this issue is not clear because of the insufficiency of experimental data. In the previously published case reports, it has been thought that OP may cause oxidative stress, giving direct damage to renal tubules and renal parenchyma, leading to dehydration due to hypovolemia, and causing development of acute renal failure. In addition, it has been stated that myoglobinuria occurring due to rhabdomyolysis caused by muscle fasciculations may contribute to the development of acute renal failure [9,28,29]. In our study also, we observed histopathological changes both in tubular structure, and glomerulus and Bowman capsule. These results suggest that OP may cause acute renal failure rather by renal parenchymal and tubular damage.

Acute tubular necrosis and intensive tubular destruction were found in the autopsy of a 68-year-old male patient who took OP for suicidal attempt and developed respiratory distress syndrome and acute renal failure [30]. In our study, in the histopathological examination performed on the sections prepared with H&E method, epithelial cell disorganization in tubules, expansion of tubules, vacuolization of tubular epithelial cells, and tubular structure approaching atrophy were observed. Remarkably, histological examinations on the sections prepared with PAS method showed the presence of intensive PAS**-**positive cytoplasmic granules in the cytoplasms of the cells forming the proximal tubules. Brush border structures with impaired continuity and losses were observed in the proximal tubule cells. These findings suggest that acute tubular damage may play a more important role in the development of acute renal failure after OP exposure.

In a study by Eid et al. with rats, Ekalux which is an OP was given to the rats for 10 days, and the changes it made in the kidney were examined under the electron microscope. As a result, they observed several changes including capillaries obstructed by the degenerated cell remains in the glomerulus, irregular wrinkling and branching, dilatation in endoplasmic reticulum in the proximal tubule cells, glycogen granule accumulation, pyknotic nucleus, electron lucency in the distal tubules, vacuolation of cytoplasm, edematous epithelial cells on the distal curved tubule wall, bleb formation, and microvillus loss [22]. In another study, after 90 days exposure to mix OP (dimethoate, acephate, dichlorvos, and phorate), renal tubular epithelium epithelial cell swelling, vacuolar degeneration, and granular degeneration were observed [24]. In these two studies, the changes that occurred after 10 and 90 days of exposure were investigated. Our study was different from the mentioned studies in demonstrating the changes in the renal tubules and glomerulus following OP acute exposure (once 0.8 g/kg fenthion).

As is known, oxidative stress leads to LPO, which in turn causes disruption of the plasma membrane. MDA is one of the products formed with decomposition of the primary and secondary LPO products, and one of the parameters used in the monitoring of LPO [31,32]. In their study, Possamai et al. showed that acute malathion exposure causes oxidative damage in the kidneys [26]. Li et al. found higher MDA level in the group they administered mixture pesticide compared to the controls [30]. In our study also, tissue level of MDA which shows LPO was higher in the experimental group than in the control and Sham groups. These findings suggest that, one of the negative effects of OP on the kidney is increased oxidative stress, and thus LPO at the tissue level.

GSH is a tripeptide known as γ-l-glutamyl- l-cysteinyl-glycine. It is a very important low**-**molecular-weight antioxidant of non-protein thiol which is synthesized in numerous living creatures from microorganisms to humans, and found in the intracellular concentration by a high rate. It is increased in the cases of oxidative stress. GSH increased as a result of induction is involved in the cellular defense against toxic and mutagenic damage [33]. In their study, Li et al. found significantly decreased levels of GSH in the group treated with mixture insecticide compared to the control group [33]. Unlike that study, in our study, we found higher GSH level in the experimental group compared to the Sham and control groups.

## Conclusions

It should not be forgotten that one of the causes of systemic complaints linked to acute toxicity in people exposed to the OP compound of fenthion may be cellular injury to glomerular and tubular structures in the kidneys. In addition, oxidative stress and antioxidant mechanisms are activated during this process. Patients with OP intoxication should be closely followed-up for acute renal failure.
